# Supplementary material for: Twinfilin modulates tissue contractility through uncapping of capping protein in C. elegans
Source: Development. 2025 Dec 4;152(23):dev205265. doi: 10.1242/dev.205265 (PMC12746075; doi:10.1242/dev.205265)
Supplement: Supplementary information [file develop-152-205265-s1.pdf]

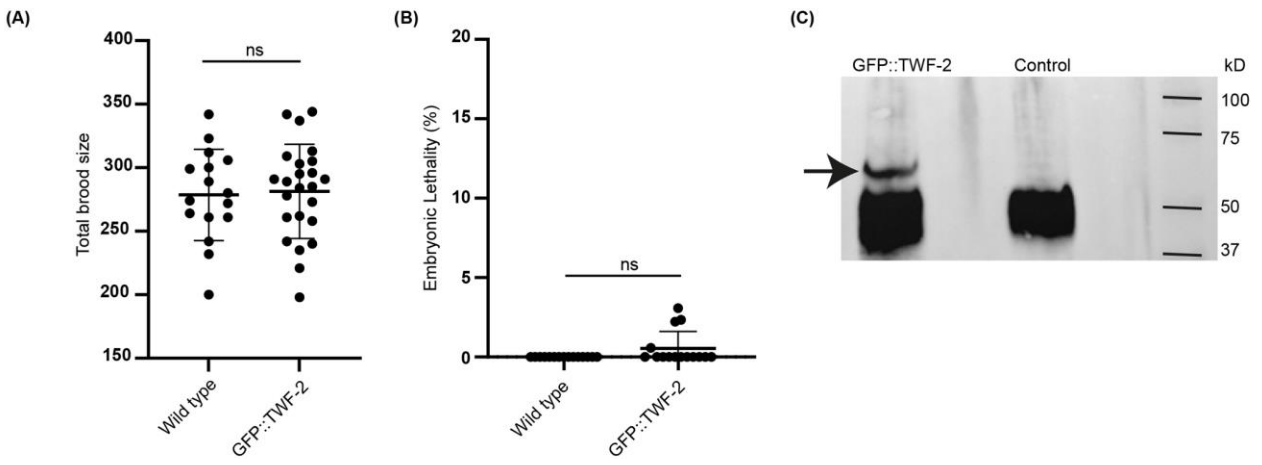

Fig. S1. Characterisation of the GFP::TWF-2 strain

(A) Brood size quantification for wild-type (N2) versus GFP::TWF-2 knock-in strains. Each point represents one hermaphrodite (N≥15 worms for each genotype). Statistical significance was determined by Mann-Whitney test (ns: not significant). Horizontal bars indicate mean ± SD.

(B) Embryonic lethality rates in wild-type versus GFP::TWF-2 animals. Statistical significance was determined by Mann-Whitney test (ns: not significant). Horizontal bars indicate mean ± SD (n ≥1000 embryos were scored for each strain)

(C) Western blot of GFP::TWF-2 and wild type control strains following an anti-GFP pulldown and stained with an anti-GFP antibody. Arrow indicates full-length GFP::TWF-2 fusion protein. Large band below is the heavy chain of the IP antibody.

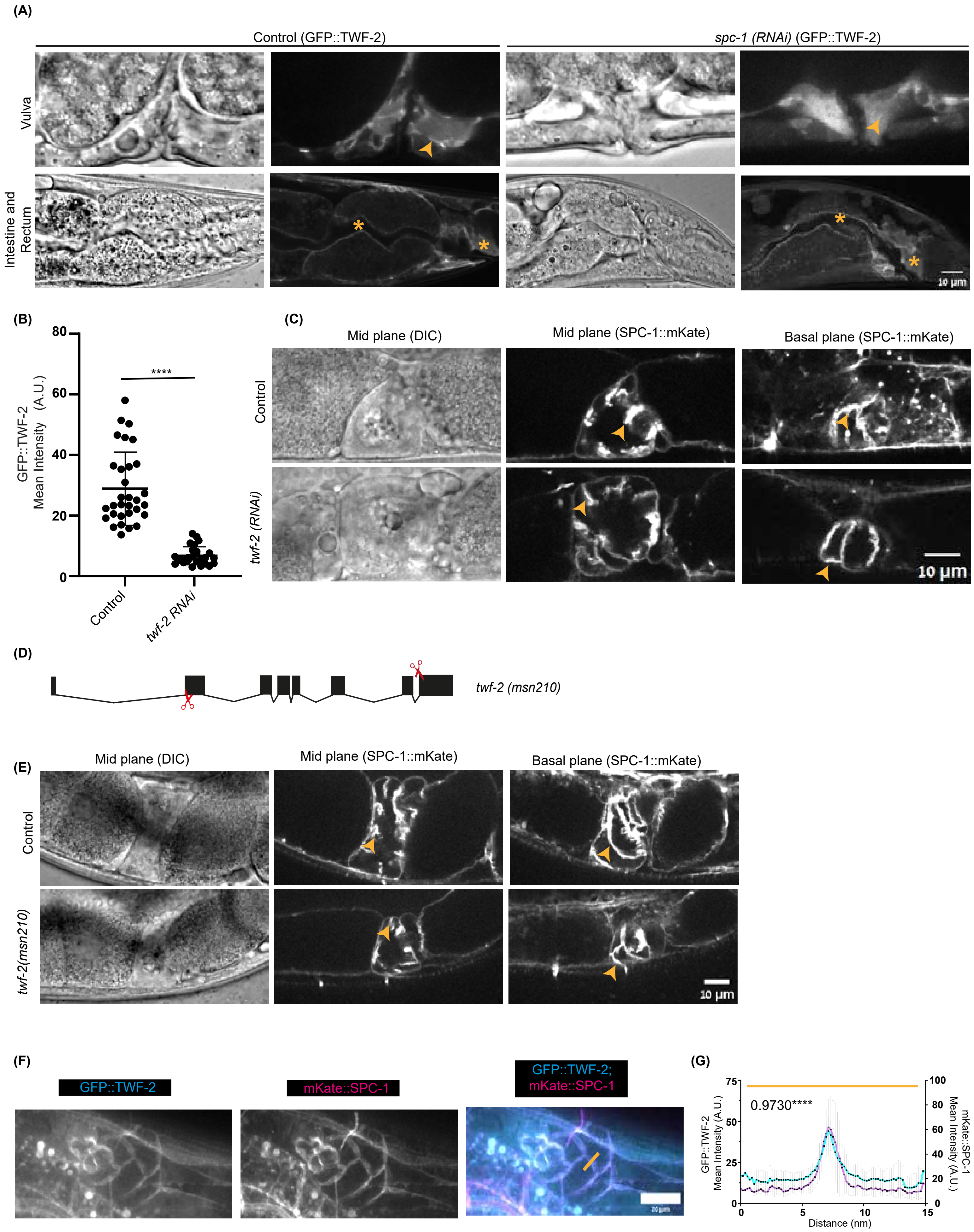

Development • Supplementary information

Fig. S2. SPC-1 regulates TWF-2 localization in a tissue-specific manner

(A) Tissue-specific effects of systemic *spc-1(RNAi)* on GFP::TWF-2 localization. Single Z-projections show GFP::TWF-2 in vulva, intestine, and rectum in control versus *spc-1(RNAi)* animals. Cortical signal is maintained (asterisks) in intestine and rectum in contrast with vulva (arrowheads) and spermatheca (Fig. 1C). Scale bar, 10 µm.

(B) RNAi efficiency quantification. Bar graph shows GFP::TWF-2 fluorescence intensity in spermathecae after control versus *twf-2(RNAi)* (****$p < 0.0001$, Mann-Whitney test). $n \geq 27$ animals were quantified for each condition.

(C) SPC-1::mKate localization is unaffected by *twf-2(RNAi)*. Representative images show spermathecal SPC-1 distribution (arrowheads) in control and *twf-2(RNAi)* animals. Scale bar, 10 µm.

(D) CRISPR-Cas9 strategy for *twf-2(msn210)* deletion allele. Schematic shows genomic organization of twf-2 locus with exons (black boxes) and introns (connecting lines). Red scissors indicate sgRNA target sites flanking the deletion region.

(E) SPC-1::mKate localization is unaffected in *twf-2(msn210)* mutant. Representative images show spermathecal SPC-1 distribution (arrowheads) in control and *twf-2(msn210)* animals. Scale bar, 10 µm.

(F) Superposition of Z-slices demonstrates spatial overlap between GFP::TWF-2 (cyan) and SPC-1::mKate (magenta) in spermatheca. Scale bar, 20 µm.

(G) Co-localization quantification for GFP::TWF-2 and SPC-1::mKate. Line profile plot shows Pearson correlation coefficient of 0.9730.

Development • Supplementary information

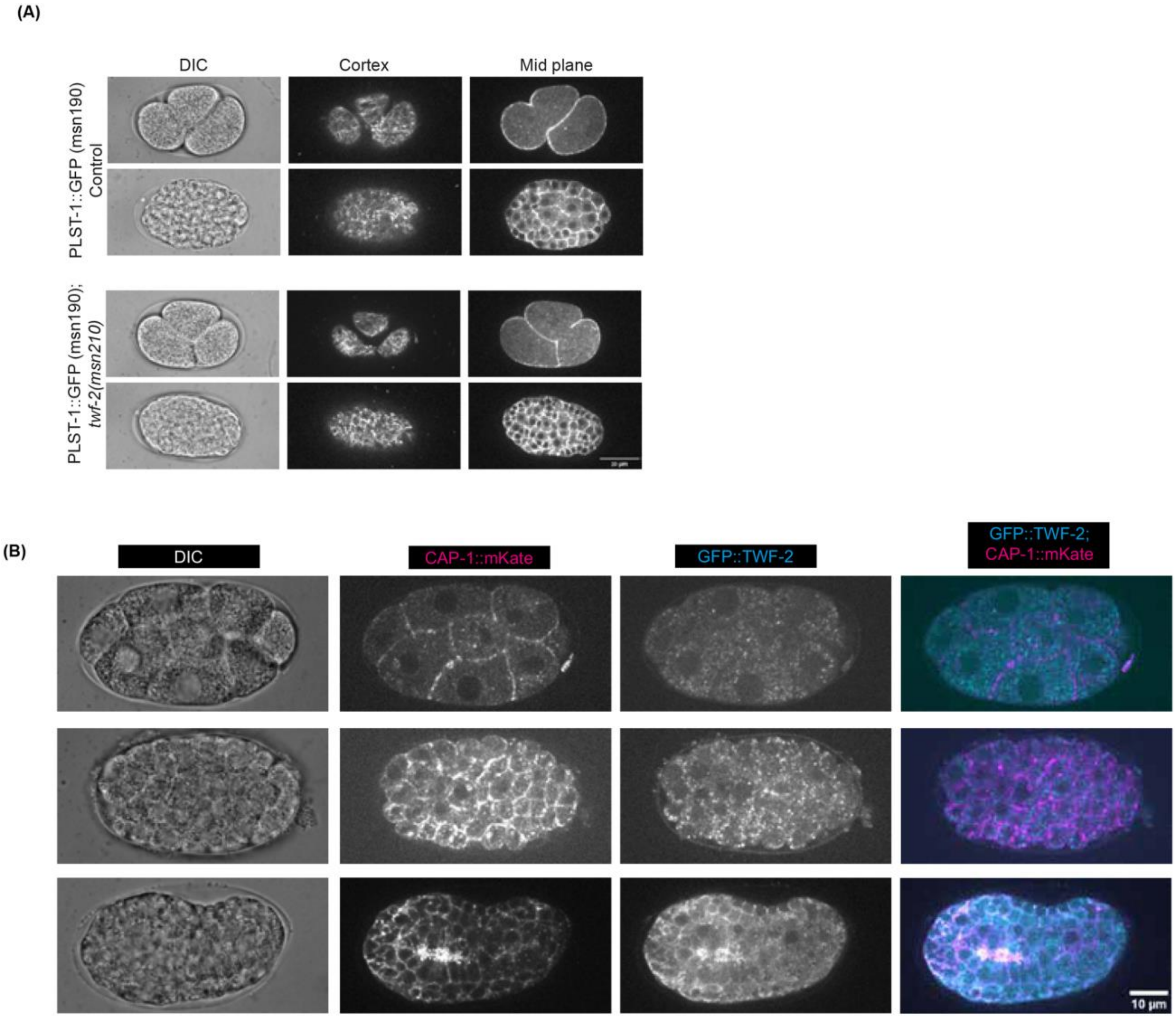

Fig. S3. TWF-2 is dispensable for actin organization and co-localizes with CAP-1 in embryos

(A) Actin organization in *twf-2(msn210)* null mutants. Representative confocal images of PLST-1::GFP-labelled actin in 3- and multi-celled stage wild-type and *twf-2(msn210)* mutant embryos. Single Z-slices show comparable actin cytoskeleton organization between genotypes.

(B) Co-localization of endogenous GFP::TWF-2 (cyan) and mScarlet::CAP-1 (magenta) in different stages of embryogenesis . Scale bar, 10 µm.

Development • Supplementary information

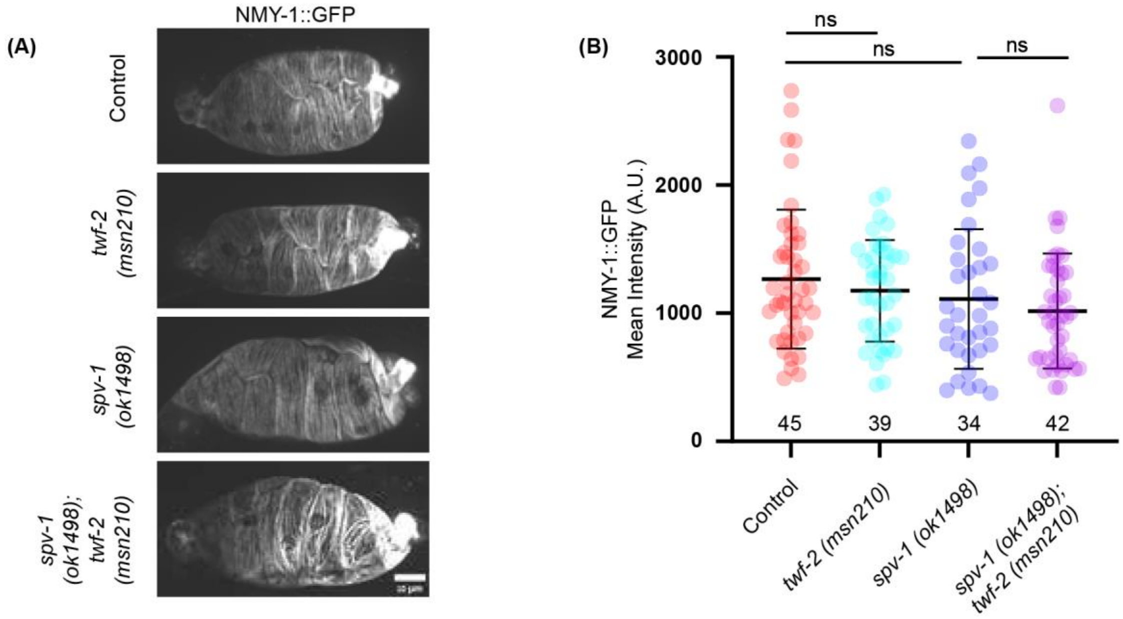

Fig. S4. Effects of *twf-2 and spv-1* mutations on endogenous NMY-1::GFP intensity.

(A) Representative maximum intensity projections of confocal images of endogenous NMY-1::GFP in control, *twf-2(msn210), spv-1(ok1498),* and *twf-2(msn210);spv-1(ok1498)* double mutant strains. Scale bar, 10 µm.

(B) Quantification of NMY-1::GFP fluorescence intensity in spermatheca. Horizontal and vertical bars represent Mean± SD. Statistical significance was determined by Kruskal Wallis test (ns: not significant). n≥34 spermathecae were scored for each genotype.

Development • Supplementary information

Table S1. List of RNAi candidates

	Gene names	Selection criteria	Readout for screening
A	*unc-27, hum-1, ule-3, arx-6, unc-52, rab-1, let-60, clik-1, ifb-2, spc-1, unc-70, unc-87, ifc-2, nmy-1, act-5, erm-1, unc-60, mlc-4*	Identified as potential cytoskeletal interactors of TWF-2 via IP-MS	GFP::TWF-2 localisation<br>Embryonic lethality with wild type and *twf-2(msn210)* mutant
B	*cyk-1, aipl-1, unc-78, cas-1, cas-2, cap-1, mel-11, gsnl-1, unc-34, cdap-2, unc-60, tth-1, mtpn-1, crml-1*	Known actin-interacting proteins; screened due to lack of phenotype in *twf-2* single mutant, assuming potential redundancy with TWF-2	Embryonic lethality with wild type and *twf-2(msn210)* mutant

Table S2. List of all strains used

Strain	Genotype	Source
N2	Wild type	CGC
RZB588	*twf-2(msn229[*GFPnovo::*twf-2]) (X)*	This study
RZB564	*dlg-1(mib23[dlg-1::mCherry])(X)*; *twf-2(msn229[*GFPnovo::*twf-2]) (X)*	This study
RZB563	*spc-1*::degron::mkate; *twf-2(msn229[*GFPnovo::*twf-2]) (X)*	This study
RZB213	*plst-1(msn190[plst-1*::GFP]) (*IV*)	Ding et al., 2017
RZB437	*plst-1(msn190[plst-1*::GFP]) (*IV*); *twf-2(msn210) (X)*	This study
RZB419	*twf-2* null*(msn210) (X)*	This study
RZB591	*cap-1(cp436*[mScarlet-I-C1::*cap-1*]) (*IV*)	CGC
RZB590	GFPnovo::*twf-2 (X)*; *cap-1(cp436*[mScarlet-I-C1::*cap-1*]) *(IV)*	This study
RZB438	*cap-1(msn207) (IV)/ tmC5*	Ray et al., 2023
RZB553	*cap-1(msn207)(IV)/ tmC5; twf-2(msn210) (X)*	This study
RZB25	*spv-1(ok1498)(II)*	Tan et al., 2015
RZB465	*spv-1(ok1498)(II)*; *twf-2(msn210) (X)*	This study
RZB333	*xbIs1611[fkh-6P::rde-1, ttx-3*::RFP]; *rde-1(ne219);spv-1(ok1498)(II)*	Kela et al., 2022
ML2540	*nmy-1(mc82[nmy-1*::GFP]) *(X)*	CGC
RZB534	*nmy-1(mc82[nmy-1*::GFP]) *(X).;spv-1(ok1498*)II (spv-1 null)	This study
RZB528	*nmy-1(mc82[nmy-1*::GFP]) *(X)*; *twf-2(msn210) (X)*	This study

Development • Supplementary information

RZB533	*nmy-1(mc82[nmy-1*::GFP]) *(X)*; *spv-1(ok1498)(II); twf-2(msn210) (X)*	This study
RZB636	*spc-1*::degron::mkate; *twf-2* null*(msn210) (X)*	This study
COP1720	*spc-1*::degron::mkate	Zaidel bar lab

Development • Supplementary information
